# Growth and safety evaluation of infant formulae containing oligosaccharides derived from bovine milk: a randomized, double-blind, noninferiority trial

**DOI:** 10.1186/s12887-014-0306-3

**Published:** 2014-12-20

**Authors:** Ferdinando Meli, Giuseppe Puccio, Cinzia Cajozzo, Giovanni Licata Ricottone, Sophie Pecquet, Norbert Sprenger, Philippe Steenhout

**Affiliations:** Dipartimento Materno Infantile, Unità Operativa di Neonatologia, Università degli Studi di Palermo, Palermo, Italy; Nestlé Nutrition, Nestec Ltd, 22 av Reller, 1800 Vevey, Switzerland; Nestlé Research Center, Nestec Ltd, Vers-chez-les-Blanc, 1000, Lausanne 26, Switzerland

**Keywords:** Infant formula, Bovine milk-derived oligosaccharides, Infant nutrition, Healthy infants

## Abstract

**Background:**

A limited number of nondigestible oligosaccharides are available for use in infant formula. This study evaluated growth and safety in infants fed formula supplemented with a mixture of bovine milk-derived oligosaccharides (BMOS). This mixture, which was generated from whey permeate, contains galactooligosaccharides and other oligosaccharides from bovine milk, such as 3′- and 6′-sialyllactose. We hypothesized that growth in infants fed BMOS-supplemented formula would be noninferior to that in infants fed standard formula.

**Methods:**

Healthy term infants ≤14 days old were randomly assigned to standard formula (control; n = 84); standard formula with BMOS (IF-BMOS; n = 99); or standard formula with BMOS and probiotics (*Bifidobacterium longum*, *Lactobacillus rhamnosus*) (IF-BMOS + Pro; n = 98). A breastfed reference group was also enrolled (n = 30). The primary outcome was mean weight gain/day from enrollment to age 4 months (noninferiority margin: −3.0 g/day).

**Results:**

189 (67.3%) formula-fed infants were included in the primary analysis. Mean differences in weight gain between the control and IF-BMOS and IF-BMOS + Pro groups were <1 g/day, with 97.5% confidence intervals above −3.0 g/day, indicating noninferior weight gain in the BMOS formula groups. Compared with control, infants in the BMOS groups had more frequent (p < 0.0001) and less hard (p = 0.0003) stools. No significant differences were observed between the control and BMOS groups in caregivers’ reports of flatulence, vomiting, spitting up, crying, fussing, and colic. When based on clinical evaluation by the investigator, the incidence of colic was higher (p = 0.01) in IF-BMOS than in control; the incidence of investigator-diagnosed colic was not significantly different in control and IF-BMOS + Pro (p = 0.15). Stool bifidobacteria and lactobacilli counts were higher with IF-BMOS + Pro compared with control (p < 0.05), whereas Clostridia counts were lower (p < 0.05) in both BMOS groups compared with control.

**Conclusions:**

Infant formula containing BMOS either with or without probiotics provides adequate nutrition for normal growth in healthy term infants. Further studies are needed to fully explore the digestive tolerance of BMOS formula.

**Trial registration:**

ClinicalTrials.gov NCT01886898. Registered 24 June 2013.

## Background

Human milk is widely recognized as the optimal source of nutrition for infants. Greater scientific understanding of the beneficial components and properties of human milk has contributed to improvements in infant formula for infants who are either partially or exclusively formula-fed. The bifidogenic properties of human milk are of particular interest, based on evidence that a bifidobacteria-predominant gut microbiota may reduce the risk of infections and allergies in infants [1-4]. Efforts to achieve similar benefits in infants who receive formula have largely focused on the addition of either probiotics or prebiotics, or both, to infant formula. Probiotics are live bacteria (often bifidobacteria and lactobacilli species) considered to have beneficial health effects [1,2]. Prebiotics are oligosaccharides that pass undigested through the small intestine in humans and are then selectively digested by potentially beneficial bacteria in the colon, such as bifidobacteria [5].

Human milk is rich in non-digestible oligosaccharides, which may play an important role in supporting the bifidobacteria-predominant gut microbiota observed in breastfed infants [6-8]. Non-digestible oligosaccharides in human milk may have other beneficial effects as well. Some human milk oligosaccharides have been shown to directly bind to pathogenic bacteria, inhibiting the attachment of these pathogens to host cells [6]. In addition, microbial fermentation of non-digestible oligosaccharides in the gut contributes to an acidic environment that may inhibit the growth of pathogens [3,5,6]. As a result of these activities, non-digestible human milk oligosaccharides are believed to play a key role in the establishment of a healthy intestinal microbiota that provides resistance to pathogen colonization [9].

Currently, the use of prebiotics in infant formulae is limited to three primary types of oligosaccharides: 1) galactooligosaccharides (GOS), which are elongations of lactose by galactose; 2) inulins, which are elongations of sucrose by fructose; and 3) fructooligosaccharides (FOS), which may be either elongations of sucrose by fructose or partially hydrolyzed inulin. In contrast, human milk contains more than 200 oligosaccharide structures that are elongations of lactose by N-acetyl-glucosamine and galactose with or without terminal fucose and sialic acid moieties [10,11]. A number of human milk oligosaccharides have been shown to have bifidogenic properties [12,13].

The lack of diversity in oligosaccharides available for use in infant formula has resulted from technical challenges in obtaining oligosaccharide structures similar to those in human milk [14]. Bovine milk contains oligosaccharides, some of which are structurally identical or similar to those found in human milk [15,16]. This suggests that oligosaccharides derived from bovine milk may provide some of the beneficial properties associated with human milk oligosaccharides. However, until recently the low concentrations of these oligosaccharides in bovine milk (approximately 20-fold lower than in human milk [14]) have hampered efforts to utilize bovine milk as a source of oligosaccharides for infant formula.

The present study evaluated infant formula supplemented with a mixture of bovine milk-derived oligosaccharides (BMOS). This mixture, which was generated from whey permeate, contained galactooligosaccharides and other oligosaccharides from bovine milk, such as 3′- and 6′-sialyllactose. Two BMOS-containing formulae were evaluated: one was supplemented with BMOS only; the other was supplemented with BMOS and the probiotics *Bifidobacterium longum* (Bl999) and *Lactobacillus rhamnosus* (LPR). The primary goal of the study was to evaluate growth and safety in infants fed the BMOS-supplemented formulae. We hypothesized that growth in infants fed BMOS-supplemented formulae would be noninferior to that in infants fed standard formula. We also explored the effects of BMOS-supplemented formulae on stooling outcomes and the composition of the gut microbiota.

## Methods

### Study design

This randomized, double-blind, single-center trial was conducted in 2007–2008 in Palermo, Italy at the Università degli Studi di Palermo, Dipartimento Materno Infantile, Unità Operativa di Neonatologia. The study was approved by the independent ethics committee of this institution and conducted in accordance with Good Clinical Practice and the principles and rules of the Declaration of Helsinki. The infants’ parents or legal guardians provided written informed consent prior to enrollment in the study.

### Population

Healthy, full-term, newborn infants were recruited from the study center during visits for routine perinatal care. Infants whose mothers had chosen to not breastfeed beyond age 14 days were randomized into the formula groups. Infants whose mothers intended to breastfeed from birth through at least age 4 months were enrolled in a nonrandomized reference group. Inclusion criteria were age ≤14 days at enrollment, weight 2500–4500 g, gestational age ≥37 weeks at birth, and singleton pregnancy. Exclusion criteria included any congenital illness or malformation that could affect normal growth, any significant pre- or postnatal disease, re-hospitalization for more than 2 days during the first 14 days of life, or antibiotic use at any time during the 5 days prior to study enrollment.

Randomization was conducted using a computer-generated randomization list with stratification by sex and delivery mode (natural or caesarian). Investigators accessed randomization numbers by logging into the computerized randomization system on a centralized server.

### Study formulae

The study formulae contained sufficient amounts of proteins, carbohydrates, fats, vitamins, and minerals for normal growth of infants from birth to age 6 months. Study formulae also contained long chain polyunsaturated fatty acids and provided 67 kcal/100 ml of reconstituted formula and 1.8 g of protein/100 kcal. The control formula was a standard, commercially available whey-based infant formula (NAN 1, Nestlé Nutrition, Nestec Ltd., Vevey, Switzerland). The two BMOS-supplemented formulae (developed at Nestlé Product Technology Center, Konolfingen, Switzerland) were similar in composition to the control formula except: a) one formula (IF-BMOS) contained BMOS at a total oligosaccharide concentration of 7.3 ± 1.0 g/100 g of powder formula (10 g/L in the reconstituted formula) replacing the equivalent amount of lactose in the control formula; and b) the other formula (IF-BMOS + Pro) contained BMOS (7.3 ± 1.0 g/100 g of powder formula) as well as the probiotics *Bifidobacterium longum* ATCC BAA-999 (Bl999) and *Lactobacillus rhamnosus* CGMCC 1.3724 (LPR) each at 2 × 10^7^ colony forming units (CFUs) per gram.

The BMOS mixture used in the formulae was derived from bovine milk whey. Briefly, an ultrafiltration permeate of bovine whey including oligosaccharides such as 3′- and 6′-sialyllactose and GOS [17] was demineralised by a combination of electrodialysis and ion exchange. Part of the remaining lactose was then enzymatically transformed into additional GOS using a fungal beta-galactosidase (Enzeco® fungal Lactase, EDC, NY). The concentration of the oligosaccharides in the final product was determined by 2-aminobenzamide labeling as described previously [18] and using laminaritriose as an internal standard.

Study formulae were manufactured, packaged in identical cans, and coded by the study sponsor. The investigator, study staff and caregivers were blinded to formula assignment throughout the study.

### Outcomes

The primary outcome was mean weight gain per day between 14 days and 4 months (112 days) of age. Secondary outcomes were mean daily length and head circumference gains from enrollment through age 4 months, measures of gastrointestinal (GI) tolerability, stool bacterial counts, and occurrence of adverse events (AEs).

Baseline data (sex, gestational age, age at enrollment, mode of delivery, APGAR scores at 1 and 5 minutes, and anthropometric measurements) were recorded at enrollment. Follow-up visits to the study center were scheduled at age 14 days and at ages 1, 2, 3, 4, 6 and 12 months. Anthropometric measurements were taken during each of these visits. Infants were weighed nude to the nearest 10 g on electronic scales calibrated according to the manufacturer’s specifications. Recumbent length was measured to the nearest 10 mm with the full body extended and feet flexed. Head circumference was measured approximately 2.5 cm above the eyebrows using a standard non-elastic plastic-coated measuring tape.

GI tolerability was assessed at each visit based on diaries caregivers kept for 3 days prior to each visit. For each of the 3 days, caregivers recorded: (1) the volume of formula intake or minutes of breastfeeding as well as intake of other foods and liquids; (2) number of stools; (3) consistency of each stool (hard, formed, soft, liquid, or watery); (4) flatulence (never, sometimes, often); (5) spitting up (never, little [≤5 ml], much [5–25 ml], very much [>25 ml]); (6) vomiting (number of episodes); (7) duration of crying (<1 hour, 1–3 hours, >3 hours); (8) fussing without crying (never, sometimes, often); (9) episodes of colic (defined as bouts of intense, inconsolable crying with painful facial expressions and pulling up of the legs); and (10) illnesses (eg, constipation, diarrhea, ear infection, eczema, fever, respiratory symptoms) and treatments (eg, antibiotics). Colic was also evaluated by the investigator at each visit and recorded as yes/no, using the following criteria: (1) paroxysms of irritability, fussing, or inconsolable crying that start and stop without obvious cause; (2) episodes lasting 3 or more hours per day and occurring at least 3 days per week for at least 1 week; and (3) no failure to thrive. Potential associated symptoms included legs drawn up towards the abdomen [19].

At age 2 months, 5 g of fresh stool were collected from formula-fed infants during the study visit. Approximately 1 g was transferred into tubes and stored at −20°C until further analysis by fluorescence *in situ* hybridization (FISH). FISH was used to quantify total bacterial counts and counts of the following bacterial species: bifidobacteria, lactobacilli, enterobacteria, clostridia, and bacteroides (performed by Microscreen, Groningen, Netherlands). Another 1 g of stool was added to a tube with 2 ml Ringer’s solution containing 10% glycerol, and then homogenized and stored at −20°C. Bl999 and LPR counts were quantified from these samples using culture plating technique (performed by ATT, Piacenza, Italy).

Blood samples (approximately 2 mL) were collected at 2 months from infants in the formula groups and analyzed for standard biochemical parameters (e.g., hemoglobin and other iron status measures, electrolytes, blood urea nitrogen). The study investigator assessed the occurrence of AEs at each visit based on interviews with caregivers. Abnormal laboratory measurements also were coded as AEs. At each visit, the investigator queried caregivers about the occurrence of respiratory tract infection, diarrhea or other GI disorders, cough, fever, skin rash, and antibiotic intake. An episode of diarrhea was defined as ≥3 loose or watery stools in 24 hours. The end of an episode was defined by two consecutive non-watery stools or no stool in a 24-hour period. Symptoms of respiratory tract infections were runny nose and chronic cough. An AE was considered serious (SAE) if it was life threatening, caused permanent harm, resulted in hospitalization or extension of in-patient hospital treatment, or was considered to be medically relevant by the investigator. The investigator assessed all AEs for relationship with study feedings. All AEs were coded using the Medical Dictionary for Regulatory Activities (MedDRA).

### Sample size

Sample size was based on demonstrating equivalence in daily weight gain between the three groups with an equivalence margin of ±3.9 g/day. However, prior to the completion of data collection, the analysis of the primary outcome was changed to a more conservative approach with a noninferiority margin of −3 g/day as recommended by the American Academy of Pediatrics (AAP) [20]. Based on the original sample size calculation, a total of 64 infants were needed in each group to detect a 3.9 g/day difference in weight gain, assuming that standard deviation [SD] = 6.1 g/day (based on a previous trial performed in Palermo, Italy [21]); α = 0.025 (due to two pairwise comparisons); and power = 0.9. With an anticipated dropout rate of 20%, the enrollment target for each group was 80 infants. This target also had adequate power to evaluate noninferiority of weight gain using the recommended margin [20] of -3 g/day. Thirty infants were enrolled in the breastfed reference group.

### Statistical methods

Baseline characteristics and AEs were analyzed in all randomized infants and all infants in the breastfed reference group. Growth, tolerance, and stool characteristics were analyzed in all infants with post-randomization data for these outcomes (primary analysis population). Anthropometric outcomes also were analyzed in a per-protocol population, which excluded infants with the following major protocol deviations: (i) life-threatening event during the study, (ii) hospitalization for >3 days, (iii) consumption of more than one bottle/week of a nonstudy formula, (iv) failure to take the assigned formulae for >3 consecutive days, or (v) discontinuation from the study before 4 months. Stool bacteria were analyzed in the subset of formula-fed infants who provided stool samples at age 2 months.

Mean weight gains (g/day) in BMOS-supplemented and control formula groups were compared using analysis of covariance (ANCOVA) correcting for sex, and the 97.5% two-sided confidence intervals (CIs) were adjusted according to Bonferroni. Weight gain was considered noninferior if the lower bounds of the 97.5% CIs for the differences in weight gain between the BMOS formula and control formula groups were above −3 g/day. Differences in mean ± SD daily gains in length and head circumference were analyzed with ANCOVA correcting for sex and reported with 97.5% CIs. All growth parameters were compared with the World Health Organization (WHO) Child Growth Standards [22].

Group differences in mean daily stool frequency were evaluated using ANOVA and adjusted for multiple testing using the Bonferroni method. Stool consistency was compared between groups using logistic regression with pair-wise comparisons adjusted for multiple testing using the Bonferroni method. Group differences in spitting up, vomiting, crying, being fussy, and having colic were evaluated using logistic regression.

Bacterial counts were log-transformed and compared between groups using the Wilcoxon rank-sum test. Counts for bacteria that could not be detected were considered to be at the lower limit of detection of 10^6^ CFU/g. P-values were adjusted for multiple testing using the Hommel method. Statistical analyses were performed using SAS version 9.1 (SAS Institute, Cary NC, USA).

## Results

### Study population

**Figure 1:**
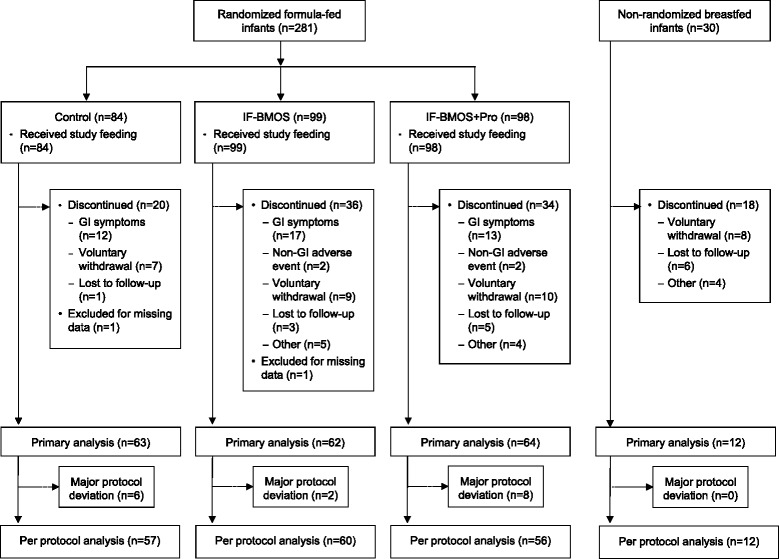
**Flow of study subjects.** GI = gastrointestinal. GI symptoms included regurgitation, vomiting, diarrhea, constipation, and abdominal pain/prolonged crying.

Three hundred and eleven healthy newborn infants were enrolled. Of these, 281 were randomized to the formula groups and 30 were enrolled in the breastfed reference group (Figure 1). Groups were balanced with respect to baseline characteristics, although the proportion of boys and caesarean births were slightly higher in the formula groups compared with the breastfed group (Table 1). A total of 90 (32%) infants from the formula groups and 18 (60%) infants from the breastfed group withdrew before the end of the study (Figure 1). Higher rates of discontinuations were observed in the BMOS-supplemented formula groups (36.4% in IF-BMOS; 34.7% in IF-BMOS + Pro) compared with the control formula group (23.8%), although the differences did not reach statistical significance (p = 0.08 for IF-BMOS versus control; p = 0.14 for IF-BMOS + Pro versus control). GI symptoms (ie, regurgitation, vomiting, diarrhea, constipation, and abdominal pain characterized by prolonged crying) were the most common reason for study discontinuation in all three formula groups: 14.3% of infants in the control group, 17.2% in the IF-BMOS group and 13.3% in the IF-BMOS + Pro group discontinued due to GI symptoms.

**Table 1:** **Infants’ baseline characteristics**

		**Control N = 84**	**IF-BMOS N = 99**	**IF-BMOS + Pro N = 98**	**Breastfed N = 30**
Sex, no. (%)	Girls	33 (39.3)	45 (45.5)	42 (42.9)	16 (53.3)
	Boys	51 (60.7)	54 (54.5)	56 (57.1)	14 (46.7)
Delivery Mode, no. (%)	Natural	33 (39.3)	43 (43.4)	41 (41.8)	14 (46.7)
	Caesarean	51 (60.7)	56 (56.6)	57 (58.2)	16 (53.3)
Gestational Age, weeks, mean (SD)		39.2 (1.1)	38.9 (1.3)	39.0 (1.3)	39.1 (1.3)
Age at enrollment, days, mean (SD)		4.5 (3.2)	5.4 (4.0)	5.0 (3.6)	5.3 (3.3)
1-minute APGAR score, median (min-max)		9.0 (6.0-10.0)	9.0 (4.0-10.0)	9.0 (0.0-10.0)	9.0 (5.0-10.0)
5-minute APGAR score, median (min-max)		10.0 (8.0-10.0)	10.0 (5.0-10.0)	10.0 (7.0-10.0)	10.0 (6.0-10.0)
Weight, kg, mean (SD)		3.3 (0.4)	3.3 (0.4)	3.2 (0.4)	3.4 (0.4)
Height, cm, mean (SD)		49.4 (1.7)	49.4 (1.8)	49.4 (1.8)	49.4 (1.6)
Head Circumference, cm, mean (SD)		34.4 (1.2)	34.3 (1.1)	34.1 (1.4)	34.4 (1.2)

Min-max = minimum-maximum; SD = standard deviation.

No significant differences in formula intake (mean daily volume) were observed among the formula groups (p > 0.05 for all comparisons). The incidence of antibiotic use during the study was comparable among the formula groups (33.3%, 30.3%, and 31.6% in control, IF-BMOS and IF-BMOS + Pro, respectively); 5 (16.7%) infants in the breastfed group used antibiotics during the study.

### Growth

Mean daily weight gain among formula-fed infants during the first 4 months of the study was between 30–32 g/day (Table 2). The mean difference in daily weight gain between each of the BMOS formula groups and the control group was less than 1 g/day, and the lower bound of the 97.5% CI of the difference in mean daily weight gain between the control and BMOS formula groups during this period was above the pre-set margin of −3.0 g/day. During the same period, infants in the breastfed group had a mean ± SD daily weight gain of 30.3 ± 5.6 g/day. Results were similar in the primary and per protocol analyses (Table 2).

**Table 2:** **Changes in anthropometric measurements between 14 days and 4 months of age**

	**Primary analysis**			**Per protocol analysis**		
	**Control N = 63**	**IF-BMOS N = 62**	**IF-BMOS + Pro N = 64**	**Control N = 57**	**IF-BMOS N = 60**	**IF-BMOS + Pro N = 56**
Weight gain, g/day, mean (SD)	30.3 (6.1)	31.6 (6.4)	30.1 (6.1)	30.2 (6.2)	31.5 (6.5)	30.5 (6.3)
Difference in weight gain compared to control, g/day, mean* (SE) [97.5% CI]		0.97 (0.97) [−1.24 to 3.17]	−0.17 (0.97) [−2.35 to 2.02]		0.94 (1.02) [−1.36 to 3.25]	0.36 (1.04) [−1.98 to 2.71]
Length gain, mm/day, mean (SD)	1.07 (0.17)	1.08 (0.19)	1.05 (0.19)	1.07 (0.17)	1.08 (0.19)	1.06 (0.20)
Difference in length gain compared to control, mm/day, mean* (SE) [97.5% CI]		0.003 (0.03) [−0.07 to 0.07]	−0.02 (0.03) [−0.09 to 0.05]		−0.01 (0.03) [−0.06 to 0.08]	−0.001 (0.03) [−0.08 to 0.07]
HC gain, mm/day, mean (SD)	0.57 (0.1)	0.56 (0.1)	0.55 (0.09)	0.58 (0.10)	0.57 (0.10)	0.56 (0.09)
Difference in HC gain compared to control, mm/day, mean* (SE) [97.5% CI]		−0.01 (0.02) [−0.05 to 0.02]	−0.03 (0.02) [−0.06 to 0.01]		−0.02 (0.02) [−0.05 to 0.02]	−0.02 (0.02) [−0.06 to 0.01]

CI = confidence interval; HC = head circumference; SD = standard deviation; SE = standard error.

*p > 0.05 for all comparisons with control.

**Figure 2:**
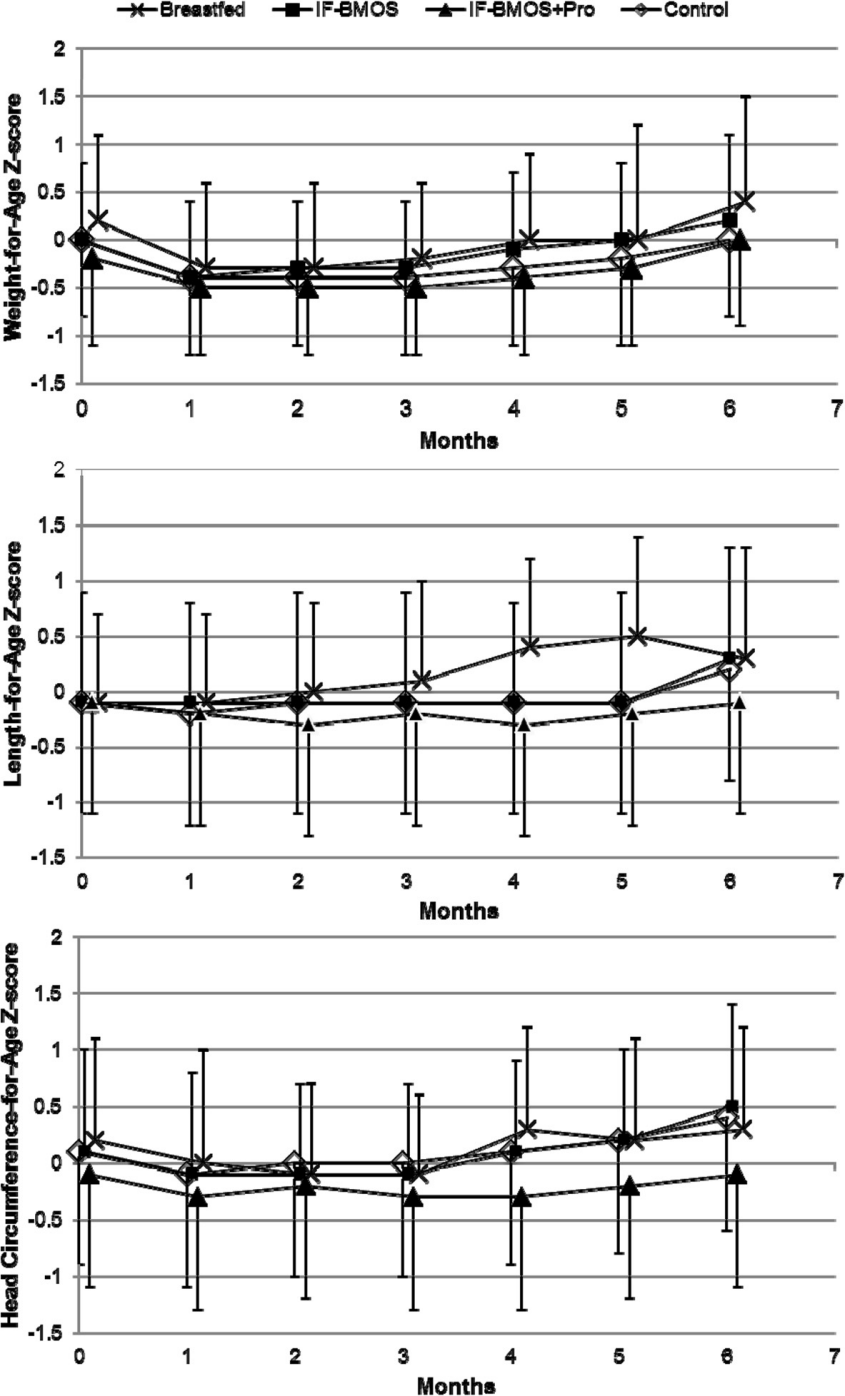
**Mean growth measurements of infants relative to World Health Organization Growth Standards.** Bars indicate standard deviations. Mean head circumference z-score at 5 months excludes the z-score of 1 infant with an implausible value (z-score = 40) at that time point only.

Mean daily gains in length and head circumference during the first 4 months showed no significant differences between the control and BMOS formula groups (p > 0.05 for all comparisons, Table 2). Compared with WHO growth standards, infants in all groups grew normally throughout the study. Mean values for all growth measures through age 4 months were within 0.5 SD of the WHO median value (Figure 2).

### GI tolerability

**Figure 3:**
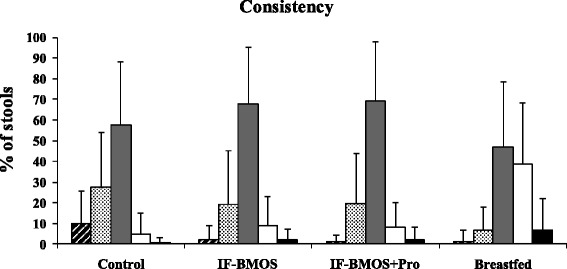
**Infant stool consistency.** Hard (hatched bars), formed (dotted bars), soft (grey bars), liquid (white bars), and watery (black bars). Bars indicate standard deviations.

Daily stool frequency in the IF-BMOS (mean ± SD, 2.6 ± 0.9 stools/day) and IF-BMOS + Pro groups (2.4 ± 0.8 stools/day) was significantly higher than in the control group (1.7 ± 0.7 stools/day, mean difference ± SE: −0.92 ± 0.13 [95% CI: −1.22 to −0.61] and −0.65 ± 0.13 [95% CI: −0.96 to −0.35], respectively, p < 0.0001 for comparisons with BMOS formula groups). Breastfed infants had 3.0 ± 0.5 stools/day. Stool consistency distributions for each group are shown in Figure 3. Infants fed the control formula were more likely to have harder stools than those fed the IF-BMOS (odds ratio [OR]: 5.06 [95% CI: 1.33 to 19.32], p = 0.0003) or IF-BMOS + Pro (OR: 6.55 [95% CI: 1.49 to 28.78], p = 0.0001) formulae.

No significant differences were observed between the control and BMOS formula groups in caregivers’ reports of flatulence, vomiting, spitting up, crying, fussing, and colic (p-values ranged from 0.19 to 0.97). However, the incidence of investigator-diagnosed colic was lower in the control group, compared with the IF-BMOS group (OR 0.38; 95% CI 0.18, 0.81; p = 0.01). The incidence of investigator-diagnosed colic was not significantly different in the control and IF-BMOS + Pro groups (OR 0.56; 95% CI 0.25, 1.24; p = 0.15).

### Stool bacterial counts

Stool samples were available from 24 (28.6%) infants in the control group, 18 (18.2%) in the IF-BMOS group, and 29 (29.6%) in the IF-BMOS + Pro group. All of the stool samples from infants in the IF-BMOS + Pro group, and approximately 80% of the samples from the control and IF-BMOS groups, had detectible bifidobacteria (Table 3). *Lactobacillus* species were detectable in nearly all of the stool samples from the IF-BMOS + Pro group. In contrast, less than 10% of samples from the control group and less than 20% from the IF-BMOS group had detectable levels of these species (Table 3). Clostridia were detected in a higher percentage of stool samples from the control group compared with either of the BMOS formula groups, and *Bacteroides* were detected in an approximately equal proportion in samples from all three groups (Table 3). Enterobacteria were detected in >95% of the samples from the control group and 100% of the samples from the BMOS-supplemented groups.

**Table 3:** **Number (%) of infants with detectable bacteria at age 2 months**

	**Control N = 24**	**IF-BMOS N = 18**	**IF-BMOS + Pro N = 29**
Total bacteria	24 (100)	18 (100)	29 (100)
Bifidobacteria	19 (79.2)	15 (83.3)	29 (100)
Lactobacilli	2 (8.3)	3 (16.7)	28 (96.6)
Enterobacteria	23 (95.8)	18 (100)	29 (100)
Clostridia	20 (83.3)	8 (44.4)	13 (44.8)
Bacteroides	5 (20.8)	3 (16.7)	5 (20.8)

Bifidobacteria and lactobacilli counts were higher in the BMOS formula groups than in the control group, though only the difference between the control and IF-BMOS + Pro groups was significant (Table 4). By contrast, clostridia counts were significantly higher in the control group compared with either of the BMOS formula groups (Table 4). Enterobacteria and *Bacteroides* counts were not significantly different between any of the groups (p > 0.1, Table 4). Bl999 was not detected in any of the samples from the IF-BMOS + Pro group, whereas LPR was detected in 16 of 29 samples.

**Table 4:** **Stool bacterial counts (log**_**10**_ **colony forming units/g) at age 2 months**

	**Control n = 24**		**IF-BMOS n = 18**		**IF-BMOS + Pro n = 29**	
	**Mean (SD)**	**Median (IQR)**	**Mean (SD)**	**Median (IQR)**	**Mean (SD)**	**Median (IQR)**
Total	10.21 (0.3)	10.29 (10.04-10.41)	10.32 (0.4)	10.32 (9.30-10.80)	10.34 (0.4)	10.44 (10.16-10.61)
Bifidobacteria	8.80 (1.7)	9.52 (7.68-10.23)	9.45 (1.8)	10.31 (9.36-10.55)	9.87 (1.2)	10.37 (9.89-10.56)*
Lactobacilli	6.13 (0.4)	6.00 (6.00-6.00)	6.27 (0.8)	6.00 (6.00-6.00)	7.68 (0.7)	7.83 (7.14-8.31)*
Enterobacteria	8.83 (0.9)	9.15 (8.58-9.31)	8.61 (0.8)	8.77 (8.06-9.03)	8.60 (0.7)	8.75 (8.31-9.03)
Clostridia	8.49 (1.4)	8.72 (7.46-9.74)	6.97 (1.3)	6.00 (6.00-7.97)*	7.01 (1.3)	6.00 (6.00-7.83)*
Bacteroides	6.37 (0.8)	6.00 (6.00-6.00)	6.30 (0.7)	6.00 (6.00-6.00)	6.48 (1.2)	6.00 (6.00-6.00)

SD = standard deviation; IQR = interquartile range.

*Significant difference compared with control (Wilcoxon rank sum <0.05).

### Adverse events

One hundred and twenty-five (45%) infants had at least one AE during the study: 36 (46%) in the control group, 39 (39%) in the IF-BMOS group, 47 (48%) in the IF-BMOS + Pro group, and 8 (26.7%) in the breastfed group (Table 5). No significant differences in the frequency of AEs were observed between groups. A total of 26 SAEs were reported in 25 infants during the 4-month intervention period. None of these were considered related to the study formulae. Hematology and blood biochemical analyses (performed in about 1/3 of formula-fed infants) were normal.

**Table 5:** **Number of serious adverse events occurring during the intervention period coded by MedDRA**

**Preferred term**	**Control n = 84**	**IF-BMOS n = 99**	**IF-BMOS + Pro n = 98**	**Breastfed n = 30**
Pneumonia	3	2	1	1
Bronchitis	2	0	2	0
Apnoea	0	1	0	0
Dyspnoea	0	1	0	0
Upper respiratory tract infection	0	0	1	0
Abdominal pain	2	0	0	0
Gastroenteritis	2	0	0	0
Diarrhea	0	1	0	0
Gastroesophageal reflux	0	0	0	1
Stupor	1	0	0	0
Convulsions	0	0	1	0
Hernia inguinal	0	1	1	0
Sudden infant death syndrome	0	0	1	0
Urinary tract infection	0	0	1	0
Total	**10**	**6**	**8**	**2**

MedDRA = Medical Dictionary for Regulatory Activities.

## Discussion

In the present study we evaluated the safety of two infant formulae containing BMOS, an oligosaccharide mixture derived from bovine milk. In general, oligosaccharides are added to infant formulae as ingredients to enhance functional properties, specifically modulation of stool frequency and consistency as well as bifidogenic and anti-pathogen properties. The oligosaccharides currently in use in infant formulae are limited primarily to GOS and FOS, and to our knowledge this is the first published report of the use of BMOS in infant formulae.

We demonstrated that infant formula supplemented with either BMOS alone or BMOS and the probiotics Bl999 and LPR met the primary safety outcome and thus provides adequate nutrition for normal growth in healthy term infants. Infants exclusively fed BMOS-supplemented formulae had weight gain similar to those fed a control formula without BMOS. The lower bound of the 97.5% CI of the difference in mean daily weight gain between the control and BMOS formula groups was above the pre-set margin of −3.0 g/day indicating noninferior growth in infants fed BMOS-supplemented formulae. Furthermore, we showed that weight, length, and head circumference measurements during the first 4 months of life were similar to WHO growth standards [20], underscoring the sufficiency of these formulae for normal growth. These results are consistent with our previous study demonstrating the safety of a synbiotic formula containing the probiotics B1999 and LPR with a combination of GOS and FOS [21]. Although the primary analysis in the present study included slightly fewer infants than the estimated number needed from the sample size calculation, it is unlikely that the addition of 1 more infant in the control group and 2 more infants in the IF-BMOS group would change the results of the analysis in a meaningful way.

The higher stool frequency observed in the BMOS-supplemented groups is similar to the effects reported in previous studies of oligosaccharides added to infant formula [21,23]. Stool frequency in the BMOS formula groups was slightly lower than in the breastfed group suggesting an effect more like that in breastfed infants. The lower frequency of hard stools in the BMOS formula groups compared with the control group may also suggest better tolerability of formula containing BMOS either with or without probiotics.

Our observation of a higher incidence of investigator-diagnosed colic in the IF-BMOS group compared with control may be due to the level of oligosaccharides added to the formula, which was somewhat higher than levels used previously [21,23]. The study did not find a statistically significant difference in risk of colic between the control and IF-BMOS + Pro formula, which suggests the possibility that the risk of colic attributable to oligosaccharides may have been modulated in a favorable direction by the addition of the probiotics. Alternatively, the lack of significance may be due to inadequate power for this particular comparison. Additional studies are planned with lower levels of BMOS.

The high number of dropouts, especially in the two test groups, may have been related in part to the higher incidence of colic and other GI symptoms in those groups, as these could have contributed to parents’ decisions to discontinue participation in the study. Although the differences in discontinuation rates between the BMOS-supplemented groups and the control group did not reach statistical significance, the study may have had inadequate power to detect such differences.

Bifidobacteria and lactobacilli were detected in a larger proportion of infants fed the formula supplemented with both BMOS and probiotics compared with those fed control formula. Furthermore, bifidobacteria and *Lactobacillus* counts were higher in infants fed the IF-BMOS + Pro formula, compared with those fed the control formula. A similar trend was observed in the IF-BMOS group with respect to a higher bifidobacteria count and a higher proportion of infants with detectable bifidobacteria compared with control; although the differences were not statistically significant. Given the exploratory nature of these analyses and the limited number of stool samples, the lack of a significant effect could be due to limited power. Furthermore, without baseline stool samples, we cannot exclude the possibility that differences in bacterial counts at baseline (e.g., due to differences in breastfeeding before day 14) was a source of confounding. However, this seems unlikely as infants were randomly assigned to the formula groups, and other baseline characteristics were balanced across these groups.

These results are consistent with a previous study that reported a bifidogenic effect of an infant formula supplemented with a mixture of GOS and FOS [24]. The present study also found lower clostridia counts in both BMOS-supplemented formula groups compared with the control group. This finding further supports the hypothesis that infant formula containing BMOS alone or BMOS with probiotics may have beneficial effects on the composition of the infant gut microbiota. Nonetheless, these results require confirmation in studies specifically focused on changes in gut microbiota as a primary outcome.

Interestingly, although bifidobacteria counts appeared slightly higher in the IF-BMOS + Pro group compared with the IF-BMOS group, the difference was not significant. This finding suggests that, at the concentration used in this study, the addition of Bl999 may not significantly increase total bifidobacteria counts above the effect of BMOS and a higher concentration may be needed to obtain an additional effect. Alternatively, collection of stools from a greater number of infants may have been needed to detect a significant difference in bifidobacteria counts between the two BMOS formula groups. Stool samples were available from only one third of the formula-fed infants, which may have limited statistical power to detect a difference between groups.

We were unable to detect Bl999 in stool from infants fed IF-BMOS + Pro, which contained this probiotic, although total bifidobacteria counts were higher with this formula compared with control. It is possible that plating method used to detect B1999 may not have been sensitive enough given the higher background level of other bifidobacteria strains. On the other hand, infants fed IF-BMOS + Pro had significantly higher lactobacilli counts compared with infants in both the control and IF-BMOS groups, presumably due to the presence of LPR (which was detected in 16/29 stool samples) in the formula. The observation that *Lactobacillus* counts were not affected by the addition of BMOS to infant formulae is consistent with the purported effect of prebiotics primarily on bifidobacteria.

An important limitation of this study is the high rate of withdrawal, which reduced the study’s power to evaluate secondary outcomes. Nonetheless, the study had adequate power to evaluate the primary outcome of the study and thus the results showing noninferiority of weight gain with BMOS-supplemented formula, as well as adequate overall growth, are robust.

## Conclusions

In conclusion, we have shown that (i) bovine milk can be used as a source of oligosaccharides for infant formula and (ii) BMOS-supplemented formula provides adequate nutrition for normal growth in healthy term infants. Further studies are needed to fully explore the digestive tolerance of BMOS formula. The addition of BMOS to infant formula resulted in more frequent, less hard stools compared with control formula; however, a higher incidence of colic was also detected. This effect was likely due to the dosage of the prebiotic and studies with lower levels of BMOS are planned. The present study also revealed positive trends in stool bacterial counts in the infants fed BMOS-supplemented formulae. Although confirmatory studies that are designed to evaluate the effects of BMOS on fecal bacteria levels are needed, these results suggest that BMOS-supplemented infant formula may be able to beneficially modulate the composition of the gut microbiota in formula-fed infants.

## Abbreviations

AAP: American Academy of Pediatrics
AE: Adverse event
Bl999: *Bifidobacterium longum* ATCC BAA-999
ANOVA: Analysis of variance
ANCOVA: Analysis of covariance
BMOS: Bovine milk-derived oligosaccharides
CI: Confidence interval
FOS: Fructooligosaccharides
GI: Gastrointestinal
GOS: Galactooligosaccharides
HM: Human milk
IF-BMOS: Infant formula supplemented with bovine milk-derived oligosaccharides
IF-BMOS + Pro: Infant formula supplemented with bovine milk-derived oligosaccharides and the probiotics *Bifidobacterium longum* and *Lactobacillus rhamnosus*
LPR: *Lactobacillus rhamnosus* CGMCC 1.3724
SAE: Serious adverse event
SD: Standard deviation
SE: Standard error
WHO: World Health Organization
